# Four lateral mass screw fixation techniques in lower cervical spine following laminectomy: a finite element analysis study of stress distribution

**DOI:** 10.1186/1475-925X-13-115

**Published:** 2014-08-09

**Authors:** Mingzhi Song, Zhen Zhang, Ming Lu, Junwei Zong, Chao Dong, Kai Ma, Shouyu Wang

**Affiliations:** 1 Department of Orthopedics, The First Affiliated Hospital of Dalian Medical University, 222 Zhongshan Road, Dalian 116011, P.R. China; 2 School of Mechanical Engineering, Dalian Jiaotong University, 794 Huanghe Road, Dalian 116028, P.R. China

**Keywords:** Finite element, Fixation, Lower cervical spine, Laminectomy, Biomechanics

## Abstract

**Background:**

Lateral mass screw fixation (LSF) techniques have been widely used for reconstructing and stabilizing the cervical spine; however, complications may result depending on the choice of surgeon. There are only a few reports related to LSF applications, even though fracture fixation has become a severe complication. This study establishes the three-dimensional finite element model of the lower cervical spine, and compares the stress distribution of the four LSF techniques (Magerl, Roy-Camille, Anderson, and An), following laminectomy -- to explore the risks of rupture after fixation.

**Method:**

CT scans were performed on a healthy adult female volunteer, and Digital imaging and communication in medicine (Dicom) data was obtained. Mimics 10.01, Geomagic Studio 12.0, Solidworks 2012, HyperMesh 10.1 and Abaqus 6.12 software programs were used to establish the intact model of the lower cervical spines (C3-C7), a postoperative model after laminectomy, and a reconstructive model after applying the LSF techniques. A compressive preload of 74 N combined with a pure moment of 1.8 Nm was applied to the intact and reconstructive model, simulating normal flexion, extension, lateral bending, and axial rotation. The stress distribution of the four LSF techniques was compared by analyzing the maximum von Mises stress.

**Result:**

The three-dimensional finite element model of the intact C3-C7 vertebrae was successfully established. This model consists of 503,911 elements and 93,390 nodes. During flexion, extension, lateral bending, and axial rotation modes, the intact model’s angular intersegmental range of motion was in good agreement with the results reported from the literature. The postoperative model after the three-segment laminectomy and the reconstructive model after applying the four LSF techniques were established based on the validated intact model. The stress distribution for the Magerl and Roy-Camille groups were more dispersive, and the maximum von Mises stress levels were lower than the other two groups in various conditions.

**Conclusion:**

The LSF techniques of Magerl and Roy-Camille are safer methods for stabilizing the lower cervical spine. Therefore, these methods potentially have a lower risk of fixation fracture.

## Background

With the steady development of internal fixation techniques, lateral mass screw fixation (LSF) with plates or rods has become the standard method for posterior cervical spine fixation and stability on various surgical indications. Many studies emphasized that LSF techniques could provide the same biomechanical stability, as compared with anterior cervical fixations or posterior wiring techniques. Additionally, due to the development of the polyaxial screw-rod system, cervical fixation surgery has now become easy to perform. Therefore, most surgeons believe the LSF techniques are the optimum methods for reconstructing the stability of the cervical spine, following decompressive surgery [1,2].

LSF techniques have been constantly studied and modified, since it was first introduced by Roy-Camille in 1972; and the techniques of Magerl, Anderson and An were extensively used as well. Even though numerous anatomical and clinical studies pointed out that the techniques of Roy-Camille and Magerl had the best practical applications, inevitable and undesirable complications still occurred, such as adjacent facet joint injury, nerve root, and vertebral artery or screw loosening and fracture [3]. In fact, all LSF techniques may result to diverse complications, depending on the surgeon’s optimization of the LSF techniques [4,5]. For the last two decades, many studies have evaluated the advantages of different LSF techniques, based on its complications [6-8]. However, we are unaware of any report that directly compares LSF techniques by analyzing fixation fractures as a severe complication -- which has a relatively low incident rate [9,10].

Due to limited case reports, it is rare find studies related to the fixation fractures of the LSF system; making it impossible to have a case analysis and difficult to simulate by traditional cadaveric experiments. A novel biomechanical approach by computer could provide a possible way to reveal the rupture risks of fixation in different LSF techniques. In recent years, finite element (FE) analysis is a technique that has been generally adopted by orthopedic experts to address biomechanical problems, especially for stress distribution [11-13]. The CT scan data obtained from a healthy volunteer was used in our study; and the four LSF techniques were applied to establish reconstructive FE models of the lower cervical spine, after a three-segment (C4-C6) laminectomy. The biomechanical stress distribution of the reconstructed models were analyzed by applying different LSF techniques via FE analysis -- aiming to provide experimental and theoretical reference for choosing the LSF technique for the lower cervical spine.

## Materials and methods

### Establishment of the intact FE model

**Figure 1:**
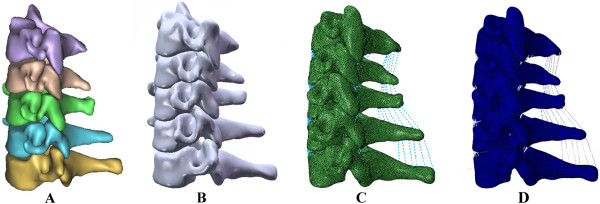
**The procedure to establish the intact 3D FE model: A: the primary model of the normal lower cervical vertebra, B: the intact model of the lower cervical spine after recovering the physiological lordosis, C: the meshed finite element of the lower cervical spine, D: the model to simulate physiological movement.**

This study was assessed and approved by the Ethics Committee of the First Affiliated Hospital of Dalian Medical University. CT scan data, with a space interval of 0.625 mm, was obtained from a thirty-year-old healthy female volunteer in an unloaded neutral position. The C3-C7 data, saved in Dicom format, was imported into the Mimics 10.01 (Materialise company, Belgium) software. A threshold was established to differentiate bone and soft tissue. Boolean calculation and interactive three-dimensional manual/automatic cutting operations were performed to establish a rough three-dimensional (3D) model of C3-C7. The model was polished, filled, and denoised by using Geomagic Studio 12.0 software (Raindrop company, USA) to smoothen uneven surfaces -- caused by stacking CT images. To reconstruct the missing physiological lordosis caused by the unloaded position, we entered the model into the Solidworks 2012 (DSSolidWorks company, USA) software to adjust the positioning of each cervical vertebra. HyperMesh 10.1 (Altair company, USA) software was used to simulate the cured surface contour of the vertebral body and intervertebral disc; then the model was meshed into a solid model. Finally, the model was imported into the FE software, Abaqus 6.12 (Simulia corp, USA), for element setup, material properties definition and FE analysis (Figure 1).

Posterior elements, cancellous bone, annulus fibrosus, nucleus pulposus, and tetrahedral elements were used for modeling vertebral bodies; cartilaginous endplates and shell elements were used for the vertebral body’s cortical bone. Ligaments were simulated as nonlinear tension-only connectors. The facet articulations of the eight pairs of zygapophyseal joints in the C3-C7 vertebrae were simulated as frictionless contact elements, due to its infinitesimal friction. The material properties of the vertebra, intervertebral disc and ligament are shown in Tables 1 and 2, according to the data reported by the reference literature [14-16].

**Table 1:** Material and mechanical properties of the different parts used for the finite element model

**Components**	**Young’s modulus (MPa)**	**Poisson’s ratio**
Cortical bone	10000.0	0.29
Cancellous bone	100.0	0.29
Endplate	500.0	0.40
Posterior structure	3500.0	0.29
Annulus fibrosus	3.4	0.40
Nucleus pulposus	1.0	0.49
Internal fixation devices (titanium alloy)	145000.0	0.30

**Table 2:** Mechanical properties of the main ligaments for the finite element model

**Deflection (mm)**	**Force ( *N *)**									
	**Anterior longitudinal**		**Posterior longitudinal**		**Spinous**		**Ligamentum flavum**		**Capsular**	
	**C3-C7**	**C5-C7**	**C3-C5**	**C5-C7**	**C3-C5**	**C5-C7**	**C3-C5**	**C5-C7**	**C3-C5**	**C5-C7**
0	0.0	0.0	0.0	0.0	0.0	0.0	0.0	0.0	0.0	0.0
1	28.0	20.0	25.0	20.0	7.0	8.0	—	—	—	—
2	52.0	40.0	44.0	40.0	12.5	14.0	38.0	30.0	55.0	75.0
3	72.0	58.0	62.0	60.0	18.0	20.0	—	—	—	—
4	89.0	78.0	78.0	78.0	22.5	25.0	60.0	68.0	130.0	145.0
5	102.0	98.0	89.0	92.0	26.0	29.0	—	—	—	—
6	115.0	112.0	—	—	30.0	32.5	80.0	102.0	180.0	204.0
7	—	—	32.5	35.0	—	—	—	—	—	—
8	—	—	—	—	—	—	108.0	130.0	210.0	250.0
9	—	—	—	—	—	—	—	—	—	—
10	—	—	—	—	—	—	—	—	230.0	265.0

### Validation of the intact 3D FE model

While the superior surface of the C3 vertebra was free, a boundary condition constraining all degrees of freedom was applied to the inferior surface of the C7 vertebra. A compressive preload of 74 N combined with a pure moment of 1.8 Nm [17] was applied on the superior surface of the C3 vertebra to simulate flexion, extension, left-right lateral bending, and left-right axial rotation movements. Abaqus 6.12 was used to validate the model by comparing the intersegmental range of motion measured from this model with the figures published by Moroney [17], Panjabi [18] and Finn [19]. If the model’s parameters are in good agreement with the normal parameters of the human body, this intact model could be used for further research.

### Laminectomy simulation and the establishment of reconstructive models

**Figure 2:**
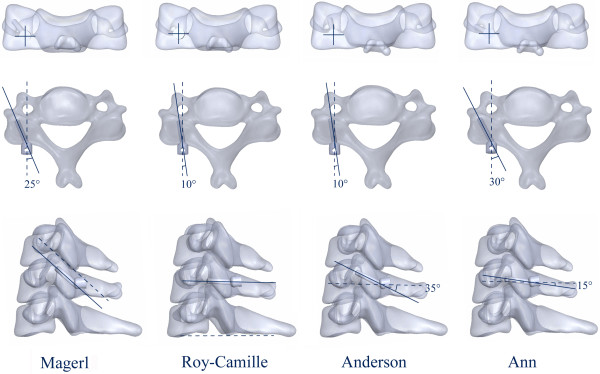
Four LSF techniques: Magerl, Roy-Camille, Anderson and An techniques.

Laminectomy was simulated in Solidworks 2012 software, which was designed to excise the lamina at C4-C6 vertebrae, the ligamentum flavum, and the spinous ligamentum at C3-C7 vertebrae. The model was built after laminectomy. 14 mm diameter screws and longitudinal connecting rods bending from 0° to 20° were designed by using the software. After laminectomy, internal fixation systems were implanted in the models at C4-C6 by applying four LSF techniques (Figure 2). During the procedure, the bone substance of the screw’s trajectory was eliminated, the screw was implanted, and each component of the internal fixation device was adjusted under direct visualization. Based on the physiological lordosis measured from the reconstructive model, we chose the bending 8° connecting rod to connect the adjacent screws.

The reconstructive models were defined as Magerl, Roy-Camille, Anderson and An groups, based on the LSF technique applied. Finally, Boolean calculation and mesh generation of the skeleton were performed, as well as the application of internal fixation devices. Elements setup, material properties definition and FE algorithms were implemented.

### Biomechanical comparison and stress analysis

The same boundary and loading conditions were applied to the four models. A compressive preload of 74 N combined with a pure moment of 1.8 Nm was applied to simulate flexion, extension, lateral bending and axial rotation. A stress distribution analysis of each fixation technique was implemented by using Abaqus 6.12.

## Results

### FE modeling and validation

**Figure 3:**
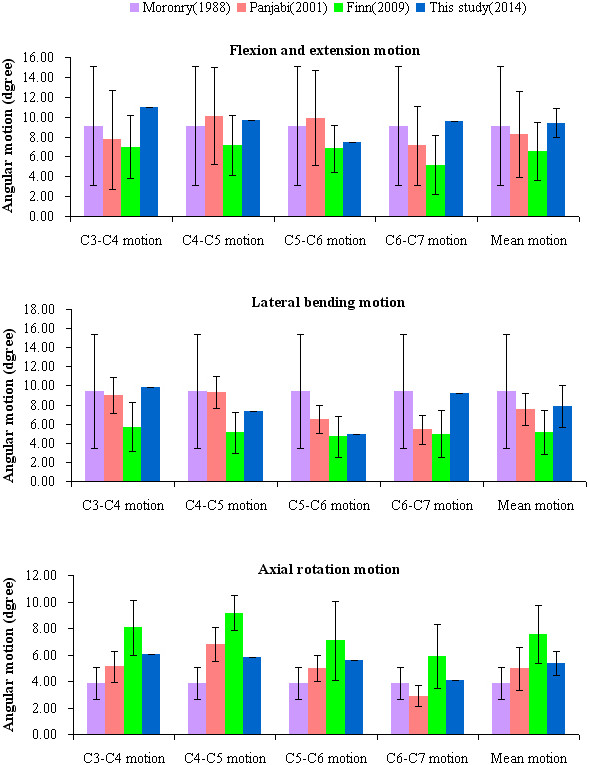
Comparison of the angular intersegmental motion, between the FE results and the results reported by the previous literature.

The intact 3D FE model of C3-C7 vertebrae was successfully established through CT scan and digital image processing, while utilizing Mimics 10.01, Geomagic Studio 12.0, Solidworks 2012, HyperMesh 10.1 and Abaqus 6.12 (Figure 1). The model contained five cervical vertebrae, four cervical intervertebral discs and five ligaments; which also consisted of 503,911 elements and 93,390 nodes (Table 3). Figure 3 shows the in vitro data details used for the comparison. The comparison of the angular intersegmental motion between the intact model and previously published data under the combined flexion, extension, left-right lateral bending and left-right axial rotation modes were summarized. There were no obvious differences (Figure 3) in the angular intersegmental range of motion between the intact model and the data published by the literature [17-19]. The data was conformed through normal human body parameters. Therefore, the intact FE model could simulate the physiological movement of the cervical vertebra, and this model can be used for our further studies.

**Table 3:** C3-C7 finite element model grid information

**Part instance**	**Element type**	**Element**	**Node**
C3	C3D4(S3)	92250	16975
C4	C3D4(S3)	89542	16509
C5	C3D4(S3)	94226	17375
C6	C3D4(S3)	105076	19333
C7	C3D4(S3)	116581	21378
Disc3/4	C3D4(S3)	2159	548
Disc4/5	C3D4(S3)	2264	596
Disc5/6	C3D4(S3)	2075	534
Disc6/7	C3D4(S3)	2358	605
Total	C3D4(S3)	503911	93390

### FE model surgery simulation

**Figure 4:**
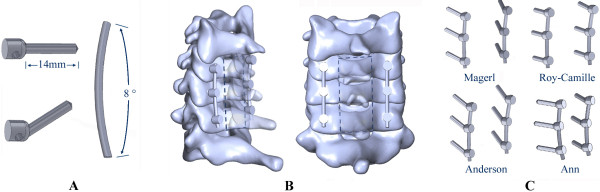
**Design process of the internal fixation device and resection and assembly process of the FE model. A**: multi-directional screws and connecting rod. **B**: resection area of the three-segment laminectomy. **C**: different fixation devices for the four LSF techniques.

**Figure 5:**
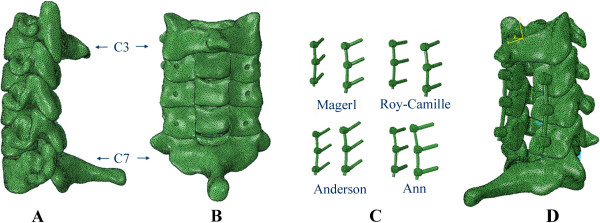
**The re-meshed model and internal fixation devices. A, B**: re-meshed FE model of the lower cervical spine following laminetomy. **C**: meshed fixation devices. **D**: re-meshed reconstructive FE model following LSF techniques.

Based on the validated intact model, the lower cervical spine model after a three-segment laminectomy and the reconstructive model applied with the four LSF techniques were established. Multi-directional screws and connecting rods were designed in Solidworks 2012. During the simulated procedure, the bone substance of the screw’s trajectory was eliminated, the screw was implanted, and each component of the internal fixation device was adjusted. The surface between the screw and the trajectory was simulated by imposing an ideal rough behavior (infinite friction coefficient). Based on the physiological lordosis measured from the reconstructive model, we chose the bending 8° connecting rod to connect the adjacent screws (Figure 4). The re-meshed models were established by using Hypermesh (Figure 5).

### Stress analysis

**Figure 6:**
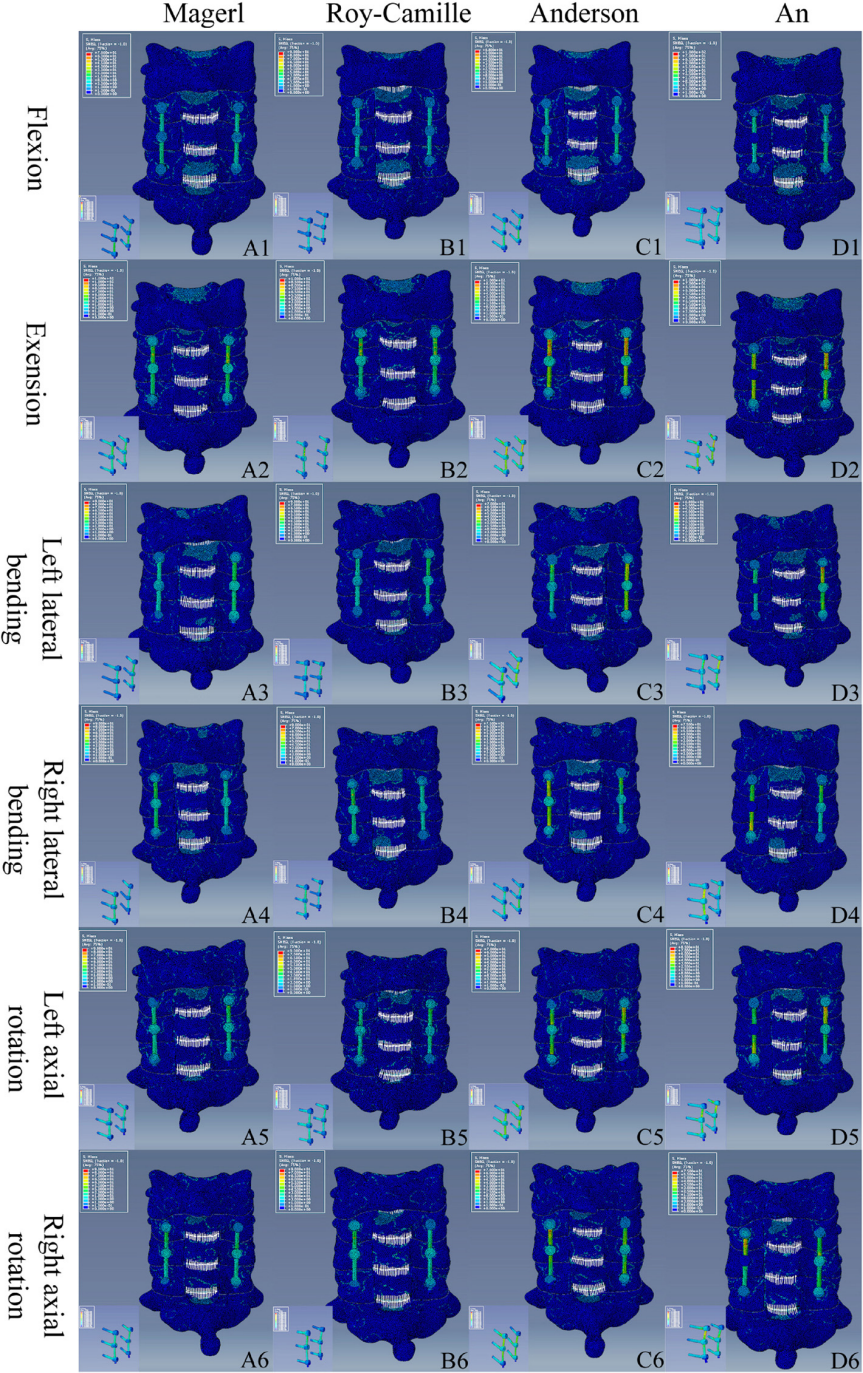
**Stress distribution of the four different LSF techniques under different conditions.** According to the indicator diagram, red area means the stress concentration, while blue area shows the stress dispersion. From **A** to **D**: Magerl group, Roy-Camille group, Anderson group and An group. From 1 to 6: flexion, extension, left lateral bending, right lateral bending, left axial rotating and right axial rotating conditions.

**Figure 7:**
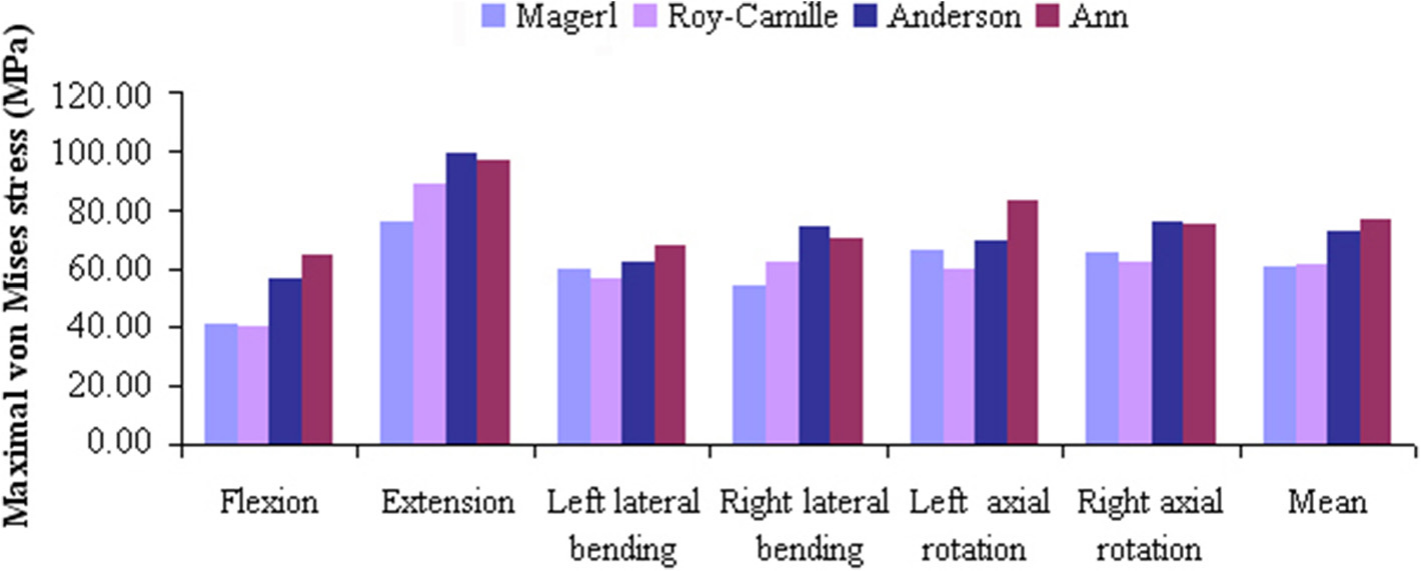
The von Mises stress of the four LSF techniques under various loading conditions.

The stress distribution on the fixation devices could demonstrate the risks of fracture, according to the fixation techniques. Figure 6 shows the stress distribution levels of the four different fixation devices during flexion, extension, left-right lateral bending, and left-right axial rotating conditions. Fixation location effects on load transfers can be evaluated based on stress concentration. Figure 7 shows the maximum von Mises stress comparisons among the fixation techniques in flexion, extension, left-right lateral bending, and left-right axial rotating conditions. For all reconstructive models, extended movement added more stress to the internal fixation system. We noted that the stress distribution in the fixation of Magerl and Roy-Camille groups were more dispersive than the other two groups (Figure 7). Higher stress concentrated areas were observed on the upper-side of connecting rod and in the rod-screw’s interface for the An and Anderson groups, especially during extension and left-right axial rotating conditions. The maximum stress level for the Anderson and An techniques in extended conditions were 99.32 MPa and 96.45 MPa, respectively (Table 4). However, the maximum stress levels for Magerl and Roy-Camille were less than 90 MPa in extended conditions. Differences were also noticeable during flexion and axial rotating conditions. In each condition, the fixation device obtained a higher level of maximum stress from the Anderson and An techniques, which indicates that Magerl and Roy-Camille LSF techniques incurs lower risks of screw fracture.

**Table 4:** The maximal von Mises stress data of four LSF techniques under various loading conditions (MPa)

**Different groups**	**Six different loading conditions**						
	**Flexion**	**Extension**	**Left lateral bending**	**Right lateral bending**	**Left axial rotation**	**Right axial rotation**	**Mean**
Magerl	41.26	75.45	59.32	54.16	66.14	65.38	60.29
Roy-Camille	40.36	88.64	56.13	62.07	59.26	62.39	61.48
Anderson	56.04	99.32	62.30	74.06	69.42	75.67	72.80
An	64.25	96.45	67.96	70.04	83.21	74.89	76.21

## Discussion

The 3D FE model is expected to provide theoretical reference for clinical practice. We established an intact model of the lower cervical spine (C3-C7) that contained bony structures, annulus fibrosus, nucleus pulposus and related ligaments (i.e. anterior and posterior longitudinal ligaments, ligamentum flavum, spinous ligament, and capsular ligaments). The intact model consisted of 503,911 elements and 93,390 nodes (Table 3), which were more elaborate than the previous models [20-23]. We simulated ligamentous structures based on the biomechanical data that was previously reported [24] and ligament functions were simulated as uniaxial nonlinear element connections. The physiological lordosis, caused by its unloaded position, recovered to accord with the authentic biomechanics of the human vertical spine. The intact model’s angular intersegmental range of motions was in good agreement with the data reported by the literature [17-19]. These results validated the model and that this model could be used for other analysis. Additionally, the mesh quantity determines the accuracy of the biomechanical analysis; our FE model promises better results in our future studies.

Based on the validated model, laminectomy was simulated to establish the postoperative model, and the four reconstructive models using the four different techniques (i.e. Roy-Camille, Magerl, Anderson, and An techniques). All simulations were performed in Solidworks 2012. With its powerful design and assembly functions, the reconstructive models were established more accurately. Fault recognition was effectively avoided, when files were exported to the other software programs – due to its software processing.

Magerl, Anderson, and An techniques were based on the Roy-Camille technique. The biomechanical stability of all four techniques could provide similar results, so as not to destroy the nucleus pulposus. In recent years, the comparisons of the above four LSF techniques mainly focused on the following aspects, facet joint violation, nerve injury, vertebral artery injury and screw pullout force. Firstly, for the facet joint violation, the point of entry and orientation of the screw were different from the four techniques. The facet joint may be violated during the screw’s implantation. Studies [25,26] have shown that approximately 50% of patients had complications due to facet joint violation, after undergoing fixation surgery with the technique of Roy-Camille. However, these complications rarely happened to patients who underwent fixation surgery using the Magerl and Anderson techniques. Secondly, for nerve injury, chronic postoperative neck pain may occur in patients after undergoing surgery with the technique of Magerl. This symptom occurs due the protrusion of the screw tip, which is close to the dorsal branch of the nerve root [27,28]. Xu et al. reported that potential risks of nerve root violation were higher in patients who underwent the Magerl and Anderson techniques, than the An technique [29]. The potential risk of nerve root injury was lower in patients who used the Roy-Camille technique, than the Magerl technique [30]. Third, for the vertebral artery injury, due to the distance of the vertebral artery from the screw’s trajectory, this artery is not likely to get injured. Heller et al. [30] found that the vertebral arteries of 26 cadaver specimens were not threatened, after undergoing LSF surgery of the lower cervical spine with the techniques of Roy-Camille and Magerl. Based on the findings of Heller, Katonis et al. [4] found no cases of vertebral artery injury among the 225 patients who underwent LSF surgery. Lastly, although comparisons had been previously conducted, the pullout force of the implanted screws, with the application of different LSF techniques, is still controversial [31]. Ulrich et al. [32] found that the imprecise fixation of screws could decrease the pullout force while undergoing surgery with the Roy-Camille technique. Magerl and An techniques paid more attention to the orientation of the screw to acquire the maximum pullout force – fixing these screws firmly to prevent extraction [25].

At present, there are still no reports on stress distribution comparisons for the four LSF techniques following laminectomy in the lower cervical spine -- for neither cadaveric study nor FE analysis. In our study, the FE analysis showed obvious differences among the four LSF techniques in response to stress distribution and maximum von Mises stress. Under various loading conditions, stress distribution in both Magerl and Roy-Camille groups were more dispersive as compared with the other two groups. The fixations in the Anderson and An groups had higher stress concentrations on the upper-side of the connecting rods and in the rod-screw’s interface. The difference of the screw’s point of entry and orientation contribute mostly to the difference in stress distribution. However, there were no significant stress distribution differences between the Magerl and Roy-Camille groups, and the Anderson and An groups. The maximum von Mises stress in the Anderson and An groups were obviously higher than the other two groups -- especially under extension and left-right axial rotating conditions. Nearly 100 MPa was concentrated on the upper side of the rod, which led to higher risks of fracture. According to the comprehensive analysis above, fixation by Magerl and Roy-Camille techniques were safer than the Anderson and An techniques. These conformed results were based on the routine choice of surgeons [3]. Although, the Magerl and Roy-Camille techniques are still better, looking from the standpoint of stress distribution.

## Conclusion

In summary, stress distribution was more dispersive and the maximum von Mises stress levels were lower in the Magerl and Roy-Camille groups for various conditions. Magerl and Roy-Camille techniques were safer for stabilizing the lower cervical spine, due to its potentially low risk of fixation fracture. We also suggest that surgeons should use new alloy materials for fixation or should limit extension movement, to retain and yield better strength and to decrease the risk of fracture, when opting for applying the Anderson and An techniques.

## Abbreviations

LSF: Lateral mass screw fixation; FE: Finite element; 3D: Three-dimensional; C3-C7: Lower cervical spine.

## Competing interests

The authors declare that they have no competing interests.

## Authors’ contributions

SMZ and MK designed this study. Both of them carried out the finite element study, analyzed the results and drafted the manuscript. ZJW helped in drafting the manuscript. DC took part in the finite element analysis. ZZ and LM helped to revise the manuscript. WSY and MK mentored the finite element analysis and provided valuable suggestions in drafting the manuscript. They were all responsible for the project and the manuscript. All authors read and approved the final manuscript.
